# 

**Published:** 1878-07-20

**Authors:** 


What will the weather be to-morrow? See advertisement.

☞ See Mark Johnson's advertisement.

Receipted.—A J Kolb, C M Bold, W A Cusick, W A Culbertson, R T Walker, 6 ms., H Pinson, T C Manning, Wm A Young, R L Seale, — Mc-Cready, 6 ms., A F Pharr, W Barton, Jno Goodman, J M Jackson, J W Rickman. J A Ardry, C C Hart, E H W Hunter, R A McIntosh, Ed Brock, R Fox, A Atkinson, J W Hoff, L G Anderson, L M Lovelace, W S Harris, G A Dyer, W C Reid, J T Suggs, B F Darnell, A J Sewell, Cochran & Kirkpatrick, J F Price, 6 ms., A G Smythe, G B Battle, E H Price. J C Anderson, J S Horsley, J G McCrary, Levi Farrow, J Horne, E G Whitman, G S Brown, 6 ms.; F. Drummond, Duke & Hudson.

